# The Comparative Effects of Atorvastatin and Quince Leaf Extract on Atherosclerosis

**DOI:** 10.5812/ircmj.4030

**Published:** 2013-08-05

**Authors:** Farzaneh Khademi, Behnam Danesh, Daruosh Mohammad Nejad, Jafar Soleimani Rad

**Affiliations:** 1 Department of Tissue Engineering and Cell Therapy, Faculty of Advanced Medical Technologies, Tehran University of Medical Sciences, Tehran, IR Iran; 2 Department of Anatomical Sciences, Faculty of Medicine, Tabriz University of Medical Sciences, Tabriz, IR Iran

**Keywords:** Atherosclerosis, Atorvastatin, Liver Enzymes, Leaf Extract

## Abstract

**Background:**

This study investigates the ability of quince leaf extract to prevent progression of atherosclerosis and to determine the lipid-lowering effect of it.

**Objectives:**

This study suggested that quince leaf effects on progression of atherosclerosis, and performed comparison with atorvastatin as a standard medication.

**Materials and Methods:**

The effect of 50mg/kg of the quince leaf extract on lipid profiles was assessed by measuring the levels of totalcholesterol, triglyceride, LDL, HDL, and liver enzymes (AST, ALT, and AP) in plasma and were evaluated the thickness of aortic plaques in the hypercholesterolemic rabbits after stopping. These assessments were performed using 0.5 mg/kg of atorvastatin.

**Results:**

Oral administration of cholesterol for 8 weeks resulted in a significant increase (P < 0.05) in plasma markers. Treatment with the extract at dose of 50 mg/kg and 0.5 mg/kg of atorvastatin not only were reduced lipid profile in plasma (P < 0.05) but also were increased HDL-cholesterol levels. There were decrease (P = 0.04) in the liver enzymes in extract treated rabbits. However, plaque thickness had no significant difference in the aorta of treated rabbits compared with studied control.

**Conclusions:**

These results indicate the lipid-lowering effects of quince leaf similar to atorvastatin and it can probably serve as a new potential natural product for atherosclerosis treatment.

## 1. Background

Advances in the medical sciences show atherosclerosis still is a major cause of cardiovascular disease and mortality in the world (1). Fatty diet, particularly LDL cholesterol, are mainly responsible for hypercholesterolemia (2) that it related to atherosclerosis (2, 3). Current atherosclerosis treatment is done using drugs or stents, atherectomy, laser angioplasty and coronary artery bypass graft in the clinic (4) to open the blocked arteries and remove plaque. Statins are cholesterol-lowering drugs which involved in the synthesis of low density lipoprotein (LDL) by enzymes such HMG-COA, leading to diminish in cholesterol levels (5, 6). But the complications of this drug are muscle toxicity called rhabdomyolysis that it can be very serious when it is taken with other lipid-lowering drugs like fibrates, particularly. Hence, using herbal drugs are significantly considered. Since plants have less toxicity effects than synthetic drugs, they have been part of medication in the past. Therefore, research on pharmaceutical plants can be a strategy for achieving new drugs with minimal adverse effects. Plants have high levels of nutritional antioxidant compounds, such as phenolic compounds (phenolic acids and flavonoids), vitamin E, carotenoids and other organic acids, which can prevent damage caused by free radicals (7, 8). Flavonoids have shown reduction in oxidation effects which lead to inhibition of atherogenesis (9-12). Hence, plants are used to reduce free radicals in the tissue damage in clinic.(12, 13) Quince tree is a good nutritional and cheap source of phenolic acids and flavonoids as antioxidants (9, 14, 15). According to the high levels of phenolic compounds in the quince leaves (such as kaempferol-phosphate), they are more effective than fruits and seeds in the promoting of health and are inexpensive source of bioactive elements (9, 15)

## 2. Objectives

This study suggested that quince leaf effects on progression of atherosclerosis, and performed comparison with atorvastatin as a standard medication.

## 3. Materials and Methods

The dry green quince leaves samples were purchased from herbs chemist. The powdered sample extracted with 70% methanol by maceration. High-fat diet with 2% cholesterol was prepared.

### 3.1. Methods

Twenty four male New Zealand white rabbits (weighing 3000 ± 200/2 g) were purchased from Pasteur Institute of Iran. Animals were kept in separate cages, under a normal pellet diet (Sahand Niroo Co, Tabriz, Iran) and also in 12 hours light and 12 hours dark conditions. They were randomly divided into normal diet (n = 6) and high cholesterol diet (n = 18).groups. Each rabbits had daily access to 100 g of pellets. Cholesterol (2 g for each animal) was dissolved in sunflower oil then given to high cholesterol diet animals by oral daily. At the end of 8^th^ week, both groups were bled of marginal vein of ear. Total cholesterol, triglyceride, HDL, LDL, aspartate transaminase (AST), alanine transaminase (ALT), and alkaline phosphatase (AP) of serum levels were measured by autoanalyzer. Then, all of the normal diet rabbits and three of high cholesterol diet group were killed and aorta was sampled to show plaque formation in histological study.

Next, the remainders of high cholesterol diet rabbits were divided into three groups of fifth after stopping fatty diet for 12 weeks: first group served as control (had not given any medication), second group was given atorvastatin (0.5 mg/kg) every day by gavage, third group fed quince leave extract (50 mg/kg) once a day by gavage. At the end of period, the aorta sampling was taken after blood sampling of ear. For sampling, animals were anesthetized by appropriate doses of Ketamine and Xylazine by intramuscular injection and histological biopsy of aorta artery was done after thoracotomy. The study was consent to the guidelines of the National Institute of Health (NIH publication 1985).

### 3.2. Biochemical Measurement and Morphometric Method

Blood samples were centrifuged at 3000rpm for 15 minutes to obtain serum for TC, TG, LDL, HDL, AST, ALT and AP. After slicing and staining with weigert iron hematoxylin, Motic Image plus2.0 software was used to measure the surface plaque.

### 3.3. Statistical Analysis

Statistical analysis was performed using the SPSS version15.0. Comparisons between two groups were performed by unpaired t-test, followed by the Man Whitney test. Significance was accepted at P-value less than 0.05. For histological data, SPSS software was used to compare mean values between the groups and P-value less than 0.01.

## 4. Results

### 4.1. Lipid Profile and Liver Enzymes Measurement

Alternation in liver enzymes and lipid profiles in blood plasma from different groups were shown in Table 1. In high-cholesterol diet group TC (P = 0.01), TG (P = 0.04), and LDL (P = 0.01) levels were significantly increased in comparison with normal diet group and HDL was decreased. Three months after stopping high cholesterol diet, lipid profiles were shown that the control group of high cholesterol diet groups did not receive any treatment after cessation of high-fatty diet, TC, TG, and LDL level was reduced compared with high cholesterol diet group which received cholesterol for two months. However, it was significantly high compared with normal diet group (P < 0.050). These profiles were significantly decreased by 0.5 mg/kg dose of atorvastatin and 50mg/kg of Quince leaf extract compared with control group of high cholesterol diet groups (P < 0.050). However, TC and LDL were highly compared with normal diet group, yet (P < 0.050). But TG and HDL levels were not shown any significant difference (Table 1).

**Table 1.** Comparison of Lipid Profile, Liver Enzymes and Plaque Thickness in Normal and High-Cholesterol Diet Groups (End of 2 Months) and in Control, Atorvastatin and Quince Leaf Extract Groups After Stopping High Cholesterol Diet (End of 3 Months)

Groups ^a^	Normal Diet	High-Cholesterol Diet ^c^	Control ^d^	Atorvastatin ^e^	Quince Leaf Extract ^f^
**TC ^b^**	76.7 23.2	2467.6 1002.1	1406.0 343.1	813.7 427.7	511.7 174.4
**TG ^b^**	179.3 14.8	1925.7 2008.2	1037.3 228.9	386.7 185.1	138.3 68.3
**LDL ^b^**	22.8 6.4	2232.8 914.6	1073.3 56.8	682.3 368.2	534.0 52.3
**HDL ^b, g^**	68.7 8.5	50.6 2.7	42.3 4.8	54.0 8.1	60.0 6.1
**AST ^b^**	50.3 6.4	83.0 7.4	86.3 10.9	45.0 4.9	45.0 7.0
**ALT ^b^**	68.0 10.1	104.6 7.5	78.0 2.0	57.7 6.0	68.7 2.7
**AP ^b^**	56.31.9	230.0 31.9	145.7 26.0	134.0 15.1	121.5 39.5
**Aorta Artery ^b^**	-	0.01 0.008	0.16 0.02	0.14 0.01	0.12 0.01

^a^ For biochemical markers, Significance was accepted at P-value less than 0.05 and for histological data P-value less than 0.01.

^c^ All biomarkers are highly than normal group (P < 0.05).

^d^ Apart from LDL, biomarkers are not meaningfully decreased in comparison with other groups and thickness of plaque are increased compared to normal group (P = 0.00)).

^e^ In TC, LDL and AP is highly than normal group (P = 0.04), but ALT reduced.

^f^ TC and LDL are increased compared to normal group (P = 0.05).

^b^ Abbreviation: ALT, Alanine Transaminase; AP, Alkaline Phosphatase; AST, Aspartate Transaminase; HDL, High Density Lipoprotein; LDL, Low Density Lipoprotein; TC, Total Cholesterol; TG, Triglyceride.

^g^ No difference in all groups.

Liver enzymes were increased significantly in high-cholesterol diet group in comparison with normal diet group (P = 0.01). After three months of stopping high cholesterol diet, the measuring of these enzymes showed that AST, ALT and AP levels were reduced in control group compared with high cholesterol diet group. But still was high than normal diet group, meaningfully (P < 0.05). AST, ALT levels in atorvastatin and Quince leaf extract groups were significantly decreased compared with control group (P = 0.04) and AP level in these studied groups were increased (P < 0.05). However, in these both groups AST and ALT were not showed any significant difference than normal diet group (Table 1).

### 4.2. Histopathological Findings of Aorta

**Figure 1.**
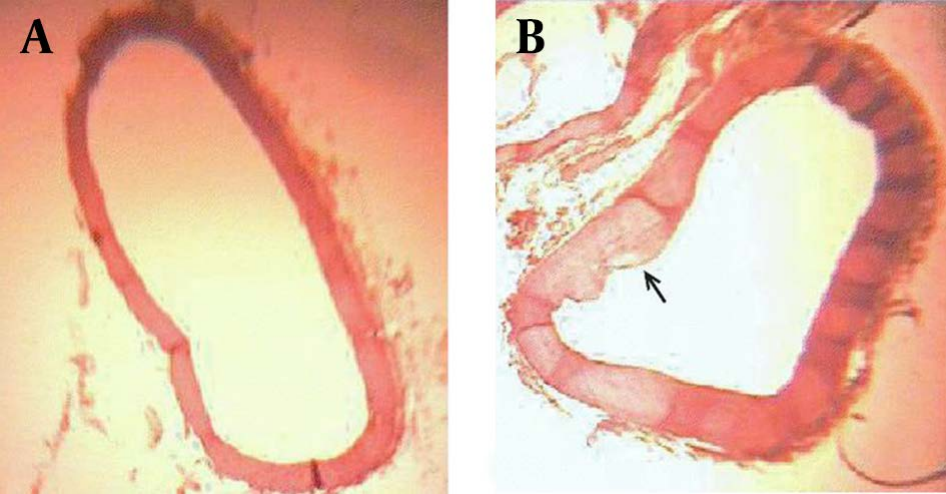
Photo micrograph of cross sectional aorta in normal diet rabbits (a) and in high-cholesterol diet rabbits (b). Thickness of atheromic plaque is shown with arrows. Weigert Iron Hematoxilin Coloration. 396-fold magnification

**Figure 2.**
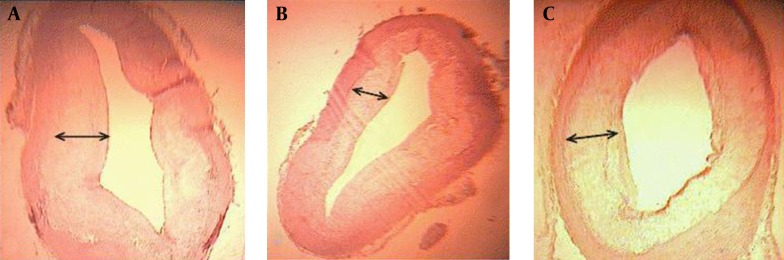
Photo micrograph of cross sectional aorta in control group of high-cholesterol diet group which did not receive any medication three months after stopping high-fat diet (a), the quince leaf extract group (b), the atorvastatin group (c). Thickness of atheromic plaque is shown with arrows. Weigert Iron Hematoxilin Coloration. 396- fold magnification

The microscopic studies of the aorta in different groups are as follows (in Table 1 and Figure 1 and 2): The cross-sectional aorta was shown in high cholesterol diet group (Figure 1 b). The plaque formation was identified as bubbles in the endothelium aorta. Plaques thickness was 0.01±0.008μm. Histological studies showed that the plaque was developed in all groups three months after the cessation of high fat diet (Figures 2 a-c). As atheromic plaque is fully expanded in control group (Figure 2 a). Plaque expansion in both quince leaf extract and atorvastatin group averagely were not significantly different compared with control group (Figure 2 b, c).

## 5. Discussion

It has been proved that one of the most important risk factor for atherosclerosis is hypercholesterolemia (1, 14). Therefore, each factor that can be reduced cholesterol levels can also be affected this process. In present study, high cholesterol level in plasma was shown by high levels of cholesterol in the diet. Similar to our result, Adaramy and Yokozawa et al. (16, 17) showed high cholesterol and triglyceride levels in rats had been received cholesterol. Also according to our results lipid profiles were return to nearly normal by standard diet for 12 weeks after stopping hypercholesterolemic diet on rabbits. Although, the histological parameters were not fully recovered (18). Moreover, we study the efficacy of quince leaf extract on atheromic plaque. Some researchers showed reduction in oxidation by flavonoids (10-12). In a research to identify the quince leaf organic compounds has been demonstrated that it contains phenolic compounds typically flavonol derivatives (19) such as having the highest amounts of kaempferol glycoside. 0-3 kaempferol Retinoid especially acts as a filter protection against UV and protects the sensitive structures such as chloroplasts (14, 15).

In our study, we showed a significant reduction in lipid profile (TC, TG, LDL) and increase in HDL cholesterol in all experimental groups after stopping atherogenic diet. Between three groups of experiment were found that the quince leaf extract group is nearest to the normal diet group. And the atorvastatin group had a better status than the control group which was not taken medication. In confirmation of our findings, reducing the profile of TC, TG, LDL and increasing HDL cholesterol levels were shown in rabbits receiving high apple juice compared with the group that had received high cholesterol. This indicates the adjustment of dyslipidemia has been effective in apple juice treat group (10). As regards, apple juice consumption has been associated with taking high cholesterol diet. Rajadurai M. in another study administrated three doses of aqueous quince leaf extract 50, 100 and 200 mg/kg with the isoproterenol (ISO) by orally and injection and compared with a-tocopherol. It was shown that the 200mg/kg dose of edible quince leaf extract could regulate the levels of lipid profiles, and LDH enzymes which were elevated by ISO. The effect of 200 mg/kg of aqueous quince leaf extract was found to equal to the effect of alpha-tocopherol 60 mg/kg. Our study demonstrated the reduction of biochemichal enzymes (increased due to fatty diet regimen) in the atorvastatin and quince leaf extract groups which these were consistent with Suk’s et al. study (20). However, the histological founding of liver damage did not improve with return to standard diet.

Assessment of histological changes: This present study indicates plaque formation in the aorta by Atherogenic Diet. The administration of 50 mg/kg dose of quince leaf extract and 0.5 mg/kg of atorvastatin for three months did not able to inhibit increasing plaque. It was higher in the control group rather than quince leaf extract group with no significant difference. Docorde et al. (21) showed that the phenols of grape, black grapes, apple juice, and apple were decreased atherosclerotic plaque in hamster by 93%, 78%, 60% and 48%, respectively. This study confirms the association of hypercholesterolemia-induced atherosclerosis in the aortic artery. The quince leaf extract similar to atorvastatin has been effective in reducing lipid profiles inducing atherosclerosis. Likely, it can be due to antioxidant components, considerably. However, according to the histological studies, both drugs did not able to prevent increasing plaque after plaque formation.
